# Public Epistemologies and Intellectual Interventions in Contemporary Italy

**DOI:** 10.1007/s10767-019-09346-3

**Published:** 2019-12-21

**Authors:** Federico Brandmayr

**Affiliations:** University of Cambridge, Cambridge, UK

**Keywords:** Science, Intellectuals, Experts, Italy, Vaccines, Anti-euro, Social media

## Abstract

Building on recent work in the sociology of intellectual interventions, the study of cultural boundaries of science, and the role of ideas in politics, the article develops a theory of public epistemologies as argumentative tools people use to support or oppose political positions. Two prominent public epistemologies that have recently crystallized in Italian politics are taken as illustrations, with special attention paid to the role of two academics (an economist and an immunologist) turned public intellectuals. The article argues that the rise of populism in Italy has contributed to unusual alignments between political and epistemological positions, which has made questions about science and expert knowledge much more relevant in contesting and supporting political decisions.

## Introduction

Science and expertise have become central political categories in contemporary democratic societies. Debates about fake news, post-truth, politicisation of research, technocracy, populist anti-intellectualism, and attacks on universities are a recurring feature of our political predicament. Instead of interpreting this either as the result of technocratic infiltration into all facets of social and political life, or as the consequences of an irrational assault on science, we should recognise that both processes are simultaneously taking place in Western democracies: an “unprecedented reliance on science and expertise” is coupled with “increase suspicion, scepticism, and dismissal of scientific findings, expert opinion, or even of whole branches of investigation” (Eyal 2019, p. 4; see also Weingart 1999, Bijker et al. 2009). In these circumstances, having the power to define what science is, what it ought to be, what it urges us to do, how we can protect it, and what its many opposites are is more crucial than ever to shape public policy as well as people’s beliefs and decisions.

This article deals with a kind of folk theorising of science that takes place in political debates. This is not limited to controversies in which the natural and medical sciences are mobilised. “Talks of science” are abound in any dispute involving sufficiently complex subjects, including economic, social, and cultural issues, as witnesses by the ubiquity of claims to expertise in debates about immigration law, economic policy, and crime control. By focusing on recent Italian politics, I argue that political debates involving complex and technical issues reveal competing public epistemologies that have a certain degree of internal consistency. Public epistemologies are recurring, complex, and relatively coherent but potentially unstable cultural schemes that define how one is to distinguish truth from falsity, what is the nature of science, what kind of people or institution can be trusted to provide reliable knowledge, and which describe in a sequential or plot-like form why ignorance and error exist in the world and what is to be done of them. Public epistemologies are systematised by intellectuals and scientists, who are increasingly engaged in highly publicised controversies. They are anchored in specific political communities and are used strategically to secure victory in deliberations over concrete policy decisions.

Where do these public epistemologies come from? How do intellectuals and experts develop, use, and disseminate them? How are they related to party platforms and political ideologies? The paper offers an answer to these question in the following way. In the first section, I review several strands of literature and introduce the concept of public epistemology to capture the relatively coherent and stable theories that actors, and particularly intellectuals, produce when talking about science. In defining this concept, I untangle my theoretical assumptions to clarify the specificities of my approach in relation to similar endeavours, such as the literature on civic epistemologies. In the second section, I present my empirical illustration, first by describing the importance of appeals to science in the Italian political context at the beginning of the twenty-first century, then by focusing on the public epistemologies elaborated by two researchers who have achieved the status of public intellectuals: an immunologist strongly engaged against anti-vaccination groups and an economist strongly engaged in favour of Italy’s withdrawal from the eurozone. In the concluding section, I extrapolate the main features of the public epistemologies found in my empirical cases, and I advance some suggestions about their connection with broader social and political transformations.

## Public Epistemologies

To think about public epistemologies, I draw on four main literatures: research on ideas in politics, and in particular on the role of “public philosophies” in shaping political debates (Schmidt 2008), research in science and technology studies on civic epistemologies (Jasanoff 2005), research on boundary work and the cultural boundaries of science (Gieryn 1999), and research on intellectual interventions and intellectual positioning (Baert 2012).

Since the 1980s, political scientists and sociologists have increasingly focused their attention on the role of (mostly economic and sociological) ideas in shaping policy-making outcomes (Campbell 2002). Ideas are generally seen by scholars as not simply reflecting the interests of voters and policy-makers but instead as relatively autonomous and as especially relevant in situations of crisis, when exogenous shocks and uncertainty disrupt established routines and force key actors to draw selectively from the available stock of cognitive tools. Scholars in this tradition ask why some ideas are more politically successful than others, and they differentiate ideas on the basis of their level of generality, from the more general level of public philosophies and world views, to the more particular level of frames and policy instruments. Public epistemologies obviously resonate with the concept of public philosophies, defined by Campbell as “broad opinions, values, and taken-for-granted cultural schema that permeate society” (1997, p. 39). But as has recently been observed, we should be careful not to underestimate public philosophies’ constantly negotiated and contested character. For example, drawing on the sociology of justification (Boltanski & Thévenot 2006) and analysing three repertoires of evaluation that have shaped French labour market policy, Carstensen and Hansen argue that such ideas “typically exhibit significant heterogeneity” and that actors actively evaluate and create compromise between them (2019, p. 598).

This is a useful insight in the study of public epistemologies. It is problematic to assign beliefs to whole groups, institutions, or societies, especially when these are traversed by deep cleavages or function according to adversarial principles. A similar argument can be advanced against certain works in the “civic epistemology” tradition (Jasanoff 2005, Miller 2008), according to which civic epistemologies include “the styles of reasoning, modes of argumentation, standards of evidence, and norms of expertise that characterize public deliberation and political institutions” (Miller 2008, p. 1896). These works tend to generalise to the national level by comparing regulatory agencies and state institutions operating in different countries. Scholars working in this tradition admit that a civic epistemology is far from being “a seamless way of knowing shared by all participants in a political community” (Jasanoff 2005, p. 231), and acknowledge that pluralist democracies “are almost inevitably characterized by a diversity of knowledge systems operating within broader civic epistemologies” (Miller 2008, p. 1899). Yet in the end their approach allows them to say, for instance, that the USA has one (“its own”) civic epistemology (Miller 2008, p. 1906). This approach also leads them to adopt a normative register, grounded in a rejection of deficit models of public understanding of science and in the willingness to engage the public in assessing the standards used by knowledge producing institutions. Civic epistemologies thus become a set of cognitive and institutional tools that can be more or less adequate to fulfil human needs for safety and wellbeing. For example, Jasanoff writes that a “nation’s civic epistemology [is] its capacity for generating reliable collective knowledge” (2005, p. 221), while Miller states that the study of civic epistemologies should reveal “how societies ensure that the public construction of epistemic authority itself conforms to accepted norms of democratic governance” (2008, p. 1905). Although these are very honourable aims, I find that such normative commitments can interfere with the goal of understanding how people think about, act on the basis of, and try to impose certain epistemological ideas in public disputes.^1^

Instead, the approach I want to defend sees public epistemologies as strategically construed and deployed in adversarial setting to influence other people and steer them toward certain decisions. As such, it owes much to the study of cultural boundaries of science as conceived in the work of Gieryn (1999), with its understanding of scientific boundary work as a credibility contest, in which players with different goals and interest compete in specific arenas “where practical decisions are negotiated and settled” (Gieryn 1999, p. 24). Scholars working in this constructivist tradition do not ask questions about the true nature or essence of science or whether a certain person, event, or object, should be considered scientific, but instead consider science as a cultural category whose meaning is, at least in certain circumstances, discursively constructed, negotiated, and contested. Although not directly focused on credibility contests, a similar and relevant perspective is Abend's programmatic outline of a sociology of epistemologies, which “investigates the epistemological bases of people’s ideas, beliefs, and understandings, and societies’ norms, practices, and institutions (ordinary people and institutions, of which scientists and science are a special part)” without focusing on truth claims per se, but on the account people make to vindicate their truth claims (2018, p. 90).^2^

Moreover, like Gieryn and others, I suggest that we should give special attention to the discourse produced by powerful actors who have the instruments to produce a complex discourse, the capacity of being heard, and the symbolic power of transforming their words into realities, such as judges, politicians, high civil servants, journalists, and intellectuals, i.e. “those who deal professionally in making things explicit and producing discourses” (Bourdieu 2005, p. 37). Researchers and experts provide sophisticated concepts, methods, and examples to build “theories of science” (Brandmayr 2018). They do so not only by intervening in the public sphere by publishing books, speaking at TV shows, writing online, or giving talks at public events, but also by deploying their expertise in a wide range of organizational settings, including state commissions, advisory boards, statistical agencies, firms, and transnational networks (Eyal & Buchholz 2010). When they narrate the vicissitudes of scientific truth, intellectuals do and achieve something: as Baert (2012) argues, intellectual interventions, including in matters epistemological, should not be conceived as representational, but as performative. By positioning themselves and others, intellectuals can transform established epistemological oppositions and alter apparently immutable cleavages. Trough intellectual interventions, public epistemologies are disseminated and they are incorporated into public discourse.

## Science and Italian Politics

**Fig. 1:**
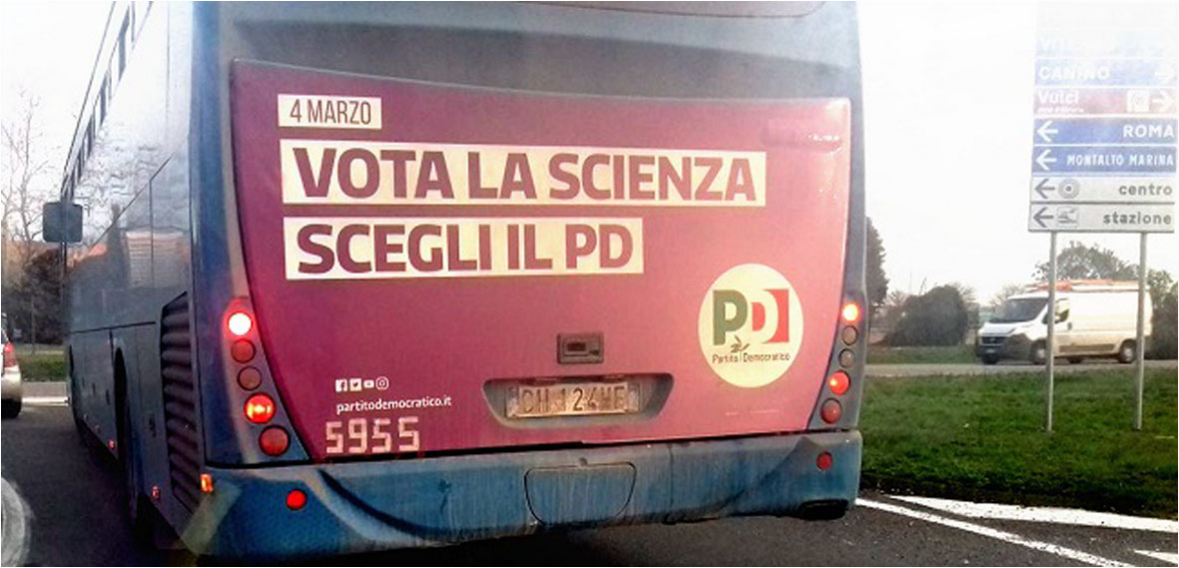
Pro-science political bus advertising in Viterbo, February 2018 (source: Biancherini 2018)

In the last decade, science has become a deeply political issue in Italy. The concept was so politically connoted that the centre-left Partito Democratico (PD) urged to “Vote for science” by “choos[ing] the PD” in one of its advertisements for the 2018 general election (see Fig. 1).

This is probably neither new nor unique to Italy. Political propaganda often presents electoral choices as highly consequential decisions in which voters have the power to protect or to endanger some important value, whether this is freedom, cultural identity, or economic growth, and science might be just another one (the PD made similar posters urging voters to choose other valuable things that the party stood for, including Europe, the environment, and culture). And that certain parties prize science more than others is also consistent with a vast literature on the politicisation of science. For example, in the USA, since the 1970s, conservatives have become more distrustful of science, while liberals’ trust remained stable (Gauchat 2012). However, various elements suggest that science has become particularly politicised in recent Italian politics, so that this country represents a strategic case to understand the development, dissemination, and use of public epistemologies.

Consider the debates that took place in the two chambers of the Italian parliament in September 2018 about the approval of a decree including a change in vaccination policy. According to the decree, parents registering their children for school could self-certify that their kids were vaccinated, as required by existing law, instead of providing official documents produced by medical institutions. For the opposition, mainly formed by centre-right and centre-left parties, this allowed parents who were hostile to vaccination to easily circumvent the obligation by lying on the self-certification, thus putting at risk the lives of children who cannot be vaccinated for medical reasons. Deputies opposing the decree called it an “attack on science” and “a declaration of war against science”. The government was accused of having chosen “superstition over science”, and of having written the decree under the advice “of shamans, not of scientists”. Members of the opposition claimed to be defending “the Italy of science, the Italy of modernity”. They said that the parliament should be united in “adopting decisions dictated by science, by good sense, which is prior to politics”, and that “on science, either you are for or against”, as “the scientific method is one and only one”. The names of several “great Italian scientists”, such as Giordano Bruno and Galileo Galilei, were invoked time after time. Members of the majority, formed by anti-establishment parties, reacted by accusing the opposition of “scientocracy”, others of transforming science into “an electoral instrument” or into an instrument to promote special interests instead of the welfare of all. A senator of the majority expressed concerns with the dichotomy that the opposition was drawing between science and politics, and claimed that it would be worrying if the latter “had no right in questioning certain absolute truths” posed by the former. He suggested that the diversity of policies that have been variably considered as scientific is proof that “science can become not the exercise of doubt and research, but the simple continuation of politics by other means”. In turn, the opposition argued that criticising the decree “did not mean that science is infallible, but simply that the fallibility of science can be ascertained only by science itself”. They added that “considering science itself as a power that is separate from the interest of the citizens” is “an obscurantist principle”. That these epistemological arguments did not apply only to vaccination policies was made clear in the speech given by a member of the opposition, a professor of economics. After having stated that the government was lingering with “the constituency of those who oppose scientific truths”, i.e. of those according to which statements are true “not because they are scientifically demonstrated, but because many people say that they are true”, he added that “this is true for vaccines but perhaps even more for economics, where in the last years prominent members of the government have championed economic theories that not only have no scientific validity, but no empirical evidence either”. Conversely, a senator of the majority, also a professor of economics, argued that one should not idolise the truths of science, offering as proof his belonging to “a scientific community that has defended austerity policies in the past and that has now changed its mind”, after having negatively affected the lives of many people.^3^

In sum, the Italian parliament was deeply divided in September 2018 about how to define science and its role in society. These debates epitomise two broad public epistemologies that exist in contemporary Italian politics. I call the first the *science aversion* narrative, and the second the *science perversion* narrative. What both narratives have in common is that they consider the place and role of science as something crucial to understand contemporary society.^4^

According to the first, Italy is a country characterised by a deep and irrational aversion to scientific and expert knowledge. Science aversion is at times framed as a betrayal and at other times as simple indifference, but the key theme is similar: the country regularly ends up being administered by people with low intellectual capacities, who disdain experts and intellectuals, believe in conspiracy theories and other pseudo-scientific ideas, and prize football, motors, and sex parties over knowledge and culture. The effects of this form of anti-intellectualism are catastrophic: measures are not taken to protect people from risks and uncertainties, to increase the efficiency of state services, or to discover new solutions to pressing social problems. Instead, politicians pass legislation that is based on emotions rather than reason, putting peoples’ lives at risk and creating deep injustices.

Science aversion narrators use a list of tropes drawing from well-known cases: the widespread belief around mid-2000s in the chemtrail conspiracy theory, leading to several parliamentary interpellations (Bianchi 2017a); the controversy around the Stamina therapy, a treatment without scientific validation aimed at neurogenerative diseases which underwent costly clinical testing in 2013 following protest by patient groups (Cattaneo & Corbellini 2014); the conviction (later overturned) of six earthquake scientists in 2012 for having downplayed the risk of an earthquake in L’Aquila, often framed as a sentence sending them to jail for not having predicted the deadly earthquake that hit the city in April 2009 (Benacchio 2012); low spending and frequent budget cuts for universities and research organisations, illustrated by common headlines such as “Italy at the tail-end [*fanalino di coda*] in research and development spending” (Marcelli 2014) or “Italian election leaves science out in the cold” (Abbott 2018).

People embracing this narrative have made the practice of “debunking” or “blasting” conspiracy theories and hoaxes (*bufale*) in social media websites something enjoyable and fashionable. They have introduced a whole series of epithets to ridicule ignorant people, notably the sympathisers of the anti-establishment Five Star Movement (M5S), such as “*webete*” (union of web and *ebete*, a stupid person) or “*la ggente*” (a twisted version of “the people”). This narrative has also been enriched by concepts having a technical and scientific flavour, such as that of “functional illiteracy” (*analfabetismo funzionale*) or “return illiteracy” (*analfabetismo di ritorno*), generally presented as the condition of a person who can read but cannot interpret new information. Accordingly, reports attributed to the UNESCO and the OECD are frequently cited in support of the claim that over 30% of the Italian population is illiterate in this sense. People raising awareness of the omnipresence of functional illiterates often assume that their cognitive deficiency would lead them to embrace false beliefs on a wide range of different topics. In every policy domain, be it the environment, migrations, or homosexual rights, there would then be an “illiterate” position based on bogus pseudo-scientific theories. In this perspective, many believe that the chemtrail conspiracy theory is to policies concerning air pollution what the opposition to Genetically Modified Organisms (GMOs) is to agricultural policy. Between 2010 and 2020, the science aversion narrative has been particularly common in mainstream media outlets such as the *Corriere della Sera*, *La Stampa*, and *L’Espresso*. Further, it has been politically championed by the PD and by other liberal, reformist, pro-European parties, such as +Europa, Scelta Civica, and sections of Silvio Berlusconi’s Forza Italia. In recent years, these parties have gathered votes from the most highly educated and wealthier strata of Italian society (De Sio 2018, Maraffi 2018).

According to the opposite narrative, the science perversion narrative, the problem is not the fact that science is ignored or distrusted, but the fact that scientific knowledge has been used in Italy and elsewhere to support special interests and has replaced the will of the people in determining what decisions are taken. At least since the 1990s, science has become an excuse to pass elitist legislation that benefits the few against the many. Arguments offered to support this claim are diverse, but often emphasise that Italy has been ruled by several so-called “technical governments”, notably from 1995 to 1996, when Lamberto Dini led a team of economists and jurists after the fall of the first Berlusconi cabinet, and, from 2011 to 2013, when economist Mario Monti led a cabinet composed by many independent experts. If the science aversion narrative labels its opponents as populist, the science perversion narrative labels its opponents as technocrats. This comes with the accusation of exploiting the allegedly neutral authority of expertise to pursue self-interested political choices, often closely associated with industrial, financial, and military powers. Against the fetishism of facts, this narrative proclaims that “where there’s a will there’s a way” (“*volere è potere*”). Against the bureaucratic routinisation of science and its transformation in a hierarchical organisation, defenders of the science perversion narrative celebrate the self-educated citizen, the passionate activist, and the maverick genius. It resonates in sarcastic remarks addressed to academics, professionals, and higher civil servants to ridicule the emptiness behind official titles and qualifications. An example is Matteo Salvini, leader of the Lega, ironically addressing the former President of the Italian Constitutional Court as “Professor, Doctor, President, Emeritus”, and contrasting “the big professors” who pretend to criticise and explain everything with his government, formed by “humble, unexperienced, poor little boys who travel in the back seats”, but who can do more in a few months than they did in forty years (Stella 2018). This public epistemology is particularly common among the Five Star Movement (M5S), the Lega (especially since the national populist turn imprinted by Salvini in 2013), and to some extent also in far-left parties such as Liberi e Uguali and Potere al Popolo. Media outlets supporting this view include Beppe Grillo’s blog (representing the radical side), *il Fatto Quotidiano* (representing the moderate side), and *La Verità*.

During the 2010s, two intellectuals have been particularly vocal in championing these two public epistemologies in political debates. Roberto Burioni, an immunologist, has been engaged in vaccines controversies and has been a staunch defender of compulsory vaccination. Alberto Bagnai, an economist, has become the leading voice of Italian Eurosceptics and has urged a withdrawal of Italy from the eurozone. The debates to which they have taken part have different temporalities and trajectories, and have engaged different disciplines, intellectuals and publics, but in both cases public epistemologies have been advanced and contested. I focus on the period after 2010, in a structural setting dominated by online platforms emphasising user-generated content.^5^

## Roberto Burioni: Defending Science against Populist Aversion

A professor of microbiology and virology at the San Raffaele University in Milan born in 1962, Burioni has a very good publication and citation record, with over a hundred publications, an h-index of 26 and over two thousand citations as of 2019, according to the Scopus database. He became actively engaged in debates about vaccine safety and mandatory vaccination at the end of 2015, when a friend invited him to the Facebook “mom group” she had created, and in which concerns about vaccines where raging wildly. Burioni accepted his friend’s invitation to write a few explanatory posts, but was shocked to discover that the opposite was happening: “it was the mothers who were explaining vaccines to me!” People “whose sole degree was the supermarket loyalty card”, people “whose sole passed tests were the blood ones”, were telling him that vaccines, “perhaps mankind’s greatest conquest, were not only ineffective, but also extremely dangerous” (Burioni 2017, Ch. 2).

He quickly became well known to the general public, started participating in talk shows and radio programmes, addressed concerns over risks associated with vaccines in his Facebook page, and published a book titled *Il vaccino non è un’opinione: le vaccinazioni spiegate a chi proprio non le vuole capire* [“The vaccine is not a matter of opinion: vaccination explained to those who really don’t want to understand it”]. The book featured entertaining catchphrases such as “there is nothing to discuss about childhood vaccination, only to administer”, and ended with a list of “opinion-free facts” (“*I fatti senza l’opinione*”), including “vaccines are safe” and “vaccines are not a conspiracy organised by multinational corporations” (Burioni 2016a).

The book was not isolated in criticising anti-vaccines beliefs (see e.g. Mantovani 2016). Anti-vaccines or vaccine hesitancy movements, generally designated by the “No Vax” label, have been active for several years in Italy, and the country has been labelled as the “anti-vaccination capital of Europe” (The Week 2018). By and large, their concerns have been especially well represented by the M5S. Beppe Grillo’s blog has frequently hosted comments on the risk associated with vaccines and on the vested interests of politicians and pharmaceutical companies in making them compulsory. Elected representatives of the M5S have proposed various legal measures to limit their use (Matteucci 2017). Possibly as a result of these efforts, vaccination coverage declined and went below the herd immunity threshold for certain diseases. Low rates of vaccination became a major concern for the centre-left government in 2016, leading to a 2017 law increasing free compulsory vaccinations from four to 12 (a number reduced to ten in a subsequent amendment), and making them mandatory for school attendance. The decree was opposed by the Lega and the M5S and originated a new wave of protest (Casula & Toth 2018).

Burioni’s real step into celebrity occurred when he wrote a Facebook post to debunk the idea that immigrants from African countries increase the likelihood of meningitis epidemics. Concerns of this sort were seemingly common following an anomalous increase in meningitis that had been reported in Tuscany in 2015 and 2016, at a time when migrant arrivals in Italy via the Mediterranean reached a historical high. In the post, published on 31 December 2016, Burioni argued that “instead of being angry with people who are guilty of nothing, let’s remember that we have an efficacious vaccine at our disposal against this meningococcus.” In this post, Burioni stated that immigrants from Africa have nothing to do with the epidemic, defined claims to the contrary as “senseless lies”, and classify those who disseminate them as “ignorant dunces”. The post was commented and shared by thousands of users, before Burioni deleted all comments, adding the following statement:

ALL COMMENTS HAVE BEEN DELETED. I would like to make clear that this page is not a place where people who do not know anything can have a “civil debate” and discuss on an equal footing with me. It is a page where I, who have been studying these topics for thirty-five years, try to explain in an accessible way how things are. I do this for free, whereas normally my time tends to be very generously paid. Making concepts accessible requires simplification but everything I write is correct and, since I always add the relevant sources, everyone can control by himself the truthfulness of what I report. But he cannot start arguing with me. I hope to have clarified the issue: here the right to speak is given only to those who have studied, and not to the common citizen. Science is not democratic (Burioni 2016b).

“Science is not democratic” became a buzzword on social media and Burioni’s defining catchphrase. It became a rallying cry for the liberal camp, a conversation stopper that could be used against “functional illiterates”. It became a contemporary “*E pur si muove!*” and Burioni a contemporary Galileo facing a mob of hysterical ignoramuses. In his books, online publications, and media appearances, Burioni reiterated this epistemological claim. He also offered a sketchy narrative explaining the aversion toward science in contemporary societies, mainly connected to the fact that the new means of communication and information, and especially social websites, have dumbed down the discussion over scientific and technical issues and put experts and laypeople on the same level. For example, Burioni claimed that:

Unfortunately, Internet is a place where facts and opinions are mixed up and confused with each other, where all voices – regardless of their authority – are on the same level, where there is no filter, where in a discussion on fire prevention you will find the firefighter and the pyromaniac undistinguished together, where in debating whether vaccines cause autism you will find a researcher who has been studying the problem with sacrifice for a lifetime and a mature playmate who claims to be a graduate of Google university and to have discovered the origin of his son’s autism thanks to her ‘mother’s instinct’ (Burioni 2016a, Ch. 1).

For Burioni, this was not the case in the past, when people would consult an encyclopaedia written by a specialist to inquire about a technical subject. He repeatedly argued that there is only one exception to the faulty norm according to which “anyone can speak about anything”, and that is sport commentary: indeed, it never occurred to him “to listen to a football announcer who ignored the offside rule”, which leads him to conclude bitterly that “today, sport is much more respected than science” (Medicalfacts 2018).

In his second book, titled *La congiura dei somari: Perché la scienza non può essere democratica* [“A conspiracy of dunces: Why science cannot be democratic”], Burioni purports to outline a phenomenology of the dunce, consisting in sentences such as “dunces can be treated with massive doses of books”, and describes a series of science hoaxes, not limited to anti-vaccines theories. He admits that scientist can lie or go crazy, but warns that one “must have confidence in the scientific community as a whole”, because within the scientific community “a dishonest person is immediately marginalised by the facts”. He softens his elitism, claiming that although science is not democratic, “knowledge is the most democratic thing in the universe” since there are no shortcuts to acquire it, and the richest person in world, like the poorest, must eventually “open a book and work hard on it” (Burioni 2017, Ch. 16).

One could argue that it is Burioni’s ill-tempered style that made him popular, reaching over 400 k followers on Facebook and over 110 k on Twitter as of December 2019, a significant achievement in the context of Italy’s social media field, especially considering that before joining the mom group in 2015, he only had around 150 contacts on Facebook (Burioni 2017, Ch. 2). Within the national context, Burioni has become a celebrity scientist (Fahy & Lewenstein 2014) who capitalises on typical attributes associated with the scientific persona, notably exploiting unanticipated contrasts that excite the enthusiasm of the public. Burioni’s appeal seems to be built on the contrast between his professional standing, which would define him as an “egghead”, and the brutal way in which he nonchalantly taunts his opponents, which would identify him more as a thug rather than an internationally renowned expert.^6^

Burioni has also become very popular among centrist politicians. A few months before the March 2018 elections, Matteo Renzi, then secretary of the PD, offered Burioni a seat in parliament claiming that “it made sense to involve certain personalities who symbolise our struggles”. While he declined the offer, stating that his priority was doing research (la Repubblica 2018a), Burioni endorsed Renzi, stating that he was one of the rare politicians to have “sided courageously in defence of science and against witchcraft” (nextQuotidiano 2017). During the 2018 national elections, debates on compulsory vaccination have been frequent, although the issue did not feature in most electoral programmes. By and large, candidates of the Lega and the M5S claimed that while they were “in favour of vaccines” (sometimes adducing that they had vaccinated their children as evidence), they were opposed to the extension of mandatory vaccination introduced by the 2017 law, on the basis that ten mandatory vaccines are too many (Bozza 2018). The “government deal” struck by the M5S and the Lega stated that “although the goal of safeguarding individual and collective health by ensuring the necessary vaccination coverage is important, it is also urgent to address the issue of the right balance between the right to education and the right to health, protecting kids who could risk social exclusion” (by being excluded from school for not being vaccinated) (il Post 2018a). The majority put forward an amendment letting parents self-certify that their children are vaccinated in order for them to be admitted to school.

In the meantime, Burioni published another book, titled *Balle mortali. Meglio vivere con la scienza che morire coi ciarlatani* [“Deadly bullshit: better to live with science than die with charlatans”] (Burioni 2018). In November 2018, he created his own platform, a website called Medical Facts, with the aim of providing reliable information and debunking false beliefs in the medical field. In January 2019, he also launched a “Cross-cutting Deal for Science” with another immunologist, Guido Silvestri, asking politicians and prominent opinion leaders to subscribe to it. The deal prescribes, among other things, that “all Italian political formations support Science as a universal value devoid of any *political colour* which enables the progress of humanity”, and that “no Italian political formation backs or tolerates in any way pseudoscience and pseudo medicine in any form” (Medicalfacts 2019).

Burioni has been subjected to various forms of critique from different fronts. Activists of the No Vax movements have made physical threats against him, and an online petition signed by more than 100,000 users compared him to Nazi physician Josef Mengele (Change.org 2017). Several commentators criticised what they depicted as an outdated, elitist, conception of science (Il Post 2018b), while others depicted him as a hired gun for “Big Pharma”, often suggesting that he profited from patenting pharmaceutical innovations related to vaccines (Cinquegrani 2017).

Within the PD, Burioni has become a totem for most of the leadership and a polluted symbol for the radical fringes of newcomers. He represents a positive or negative symbol of how elites in general should relate with the rest of the population, and especially how political elites should relate with common citizens. In November 2018, at the PD national assembly in Rome, Dario Corallo, a young candidate to be party secretary, stated that “we have decided to tell the story of the 1% and we have humiliated the 99%, like any Burioni would do, by entertaining himself with bullying those who try to express a doubt in simple terms”. This provoked a reaction from the party leadership: under the pressure of activists and journalists asking them to take side between Corallo and Burioni, most rallied around the latter (la Repubblica 2018b).

## Alberto Bagnai: Defending Science Against Elitist Perversion

Born in 1962, Bagnai obtained a doctoral degree at the University of Rome “La Sapienza” in 1994 and has been associate professor of economic policy at the University of Chieti since 2005. Since the 2011, he has been an outspoken critic of EU economic policies and the leader anti-euro economist in the country. That Bagnai’s active engagement started in 2011 is no coincidence. Negative sentiments toward the European Union (EU) and the Eurozone have become widespread in Italy since the depths of the European debt crisis. Italy is among the countries in which the attitude toward the EU has most deteriorated in the decade from 2008 and 2018 (Debomy et al. 2018). Berlusconi’s 2011 resignation followed the rising “spread” (the difference between Italy’s 10-year bond yields and its German counterpart, reflecting investors’ fear of investing on Italian bonds) and intense negotiations with EU institutions and other countries. The European Central Bank made its support (i.e. the purchase of Italian bonds) conditional on the immediate adoption of measures of deficit reduction and labour market liberalisation. As such, the whole process (including the subsequent nomination by then-president Giorgio Napolitano of economist Monti as prime minister) has been perceived by many as foreign interference on internal affairs, or even as a “coup d’état” led by EU institutions, the IMF and powerful foreign governments, notably Germany and France (Matthijs & Blyth 2011).

Between 2011 and 2013, several authors published books claiming that the institutional structure of the Eurozone was negatively affecting Italy’s economy.^7^ These books were distributed by small and eccentric publishers, most of them of recent creation. Unable to reach a wide public by traditional publishing methods, most anti-euro authors created websites and weblogs in which they analysed the crisis as it was unfolding, targeting mostly young, male and fairly educated cybernauts. In mainstream media, the idea of leaving the Eurozone was associated with weird mavericks, for the most part devoid of any academic standing, which were depicted by high-profile economists as “denialists” and “conspiracy theorists” (Bisin 2013), while the idea of withdrawing from the Eurozone was deemed a “folly” (Pilati 2010) and “impossible” (Dadush 2011). Indeed, unfounded rumours according to which Italians had been deceived by being granted an unfavourable conversion rate from the lira to the euro were common on social networks and other media. Yet, on the other hand, journalists and “debunkers” tended to see all critiques of the euro as the outcome of ignorance of basic economic principles or simple credulity.

Bagnai entered this field as a relatively unknown professor of economics at a provincial university and positioned himself between two extremes: on the one hand, prominent orthodox economists associated with elite institutions such as Bocconi University (notably Alberto Alesina and Francesco Giavazzi) who supported EU targets of low inflation, deficit reduction, and liberalisation, and, on the other hand, amateurs with little or no academic standing who clumsily urged to burn EU institutions to the ground. Initially addressing people on the left, Bagnai argued that an anti-euro position required believing not in plots orchestrated by extravagant secret societies, but just in old-school class conflict and geopolitics. Although an entertaining and often sarcastic writer, Bagnai emphasised the importance of rigour and accuracy in making the case against the euro, citing academic sources and using datasets from trusted sources such as the IMF or national statistical agencies. Several of his blog posts are longer than many academic articles in economic journals. Bagnai’s standing as a serious thinker was also reinforced by a series of extra-academic assets: he is fluent in French, English and German, makes abundant use of Greek and Latin phrases, and plays harpsichord in several baroque ensembles.

In 2010 and 2011 wrote several articles in which he criticised the euro for heterodox economics webzines, such as *Sbilanciamoci*, for a communist newspaper, *il Manifesto*, and for liberal magazines such as *Lavoce.info*. In November 2011, when the editorial staff of *Lavoce.info* refused to publish a paper in which he criticised Monti’s contractionary fiscal policies, arguing that Italy was facing a crisis of private debt due to trade imbalances rather than a crisis of public debt, Bagnai decided to create his own platform, a BlogSpot blog titled *Goofynomics*. In 2012, his book *Il tramonto dell’euro. Come e perché la fine della moneta unica salverebbe democrazia e benessere in Europa* [“The sunset of the euro: how and why the end of the single currency would save democracy and welfare in Europe”] was published by a then-recently created publisher, Imprimatur, which has since become a prolific actor in the dissemination of contrarian and Eurosceptic ideas (Bagnai 2012b).

In 2013, Bagnai created Asimmetrie - Italian Association for the Study of Economic Asymmetries, with the aim of promoting research activities related to economic asymmetries such as those between the North and the South of the EU. The charter of the association states that “particular attention will be given to the monitoring and refutation of moralistic and partial attitudes in the divulgation of economic facts, and to the promotion of activities of popularisation based on the analysis of data and on scientific rigour, with the specific aim of creating a bridge between the frontier of scientific research and citizens’ spontaneous knowledge” (Asimmetrie 2013). In 2014, his book *L’Italia può farcela. Equità, flessibilità, democrazia. Strategie per vivere nella globalizzazione* [“*Italy Can Do It. Fairness, flexibility, democracy. Strategies to live in a globalised world*”] was published by the much more established publisher Il Saggiatore (Bagnai 2014d). Although Bagnai still has no outstanding publication record, he has published in a few highly specialized outlets, such as *Energy Policy*, and in prominent “heterodox” journals such as the *Cambridge Journal of Economics*. On Twitter, Bagnai has around 100 k followers, the only active Italian economist with a higher share being Tito Boeri, a professor at Bocconi University and former director of the national social security agency. According to the influential IDEAS database, Bagnai ranks 19th among economists on Twitter by number of followers (IDEAS 2019). From October 2012 to February 2019, Bagnai has tweeted over 183,000 times, averaging 80 tweets a day. As of December 2019, he has published almost 2000 posts on his blog, which have been visited almost fourty million times.

At the beginning of his public activity, Bagnai was not siding for any party, because none saw the euro as a real problem. He quickly became dissatisfied with the ambivalence of the M5S, especially for its anti-statist attitude (Bagnai 2014c) and its proposal of organising a referendum to leave the Eurozone (for Bagnai, only a secret and rapid withdrawal would preserve Italy from capital flight) (Bagnai 2012a). He also progressively distanced himself from Eurosceptic representatives of the left, such a Stefano Fassina, whom he accused of backing what he considered to be the shallow reformism of the Greek Syriza, the Spanish Podemos, and the French Left Front (Bagnai 2015). Bagnai exemplified his disenchantment with the left by referring to the 13 November 2011 front page of the daily *l’Unità*, founded in 1924 by Antonio Gramsci, in which Berlusconi’s resignation (and, in Bagnai’s interpretation, even Monti’s subsequent EU-led appointment) were represented as a “liberation” (2014b). Eventually, Bagnai concluded that Salvini’s Lega Nord was the only party that truly sided with the working class and that defended Italian sovereignty against foreign interference. In 2015, still identifying as a “progressive Keynesian”, he started welcoming the possibility of being the minister of the economy in a future government led by Salvini (Cerami 2015). Just before announcing his candidacy with the Lega, he wrote that “there is nothing new: it’s all written in [Jean-Claude] Michéa’s books, including the fact that there are historical phases in which, in order to defend ‘left-wing ideals’, it is first necessary to destroy the left” (Bagnai 2018). On 23 January 2018, Salvini held a press conference at the Chamber of Deputies in which he said that Bagnai’s *The Sunset of the Euro* “had opened up a whole new world” for him and had brought the Lega “to embrace a certain type of economic and cultural battle”. On 4 March 2018, Bagnai was elected to the Senate.

With regard to his public epistemology, Bagnai seems to manifest a certain ambivalence between intellectualism and anti-intellectualism that is not present in Burioni’s case. On the one hand, Bagnai has regularly emphasised the importance of scientific methods and of the consensus within the community of professional economists, and has made abundant use of certain markers of epistemic legitimacy. In blog posts, interviews, and his books, he has frequently remarked that the views he was defending were not really his, but rather the expression of a consensus of economists dating back at least fifty years. The main reasons why the euro is not a good institutional arrangement are to be found in textbooks and works written by authoritative thinkers who have been recognized as such by winning Nobel prizes, publishing in first-class journals, and occupying positions of leadership in scientific associations. In an early blog post, anticipating that readers may have been sceptical of his ideas and consider him as “the usual visionary blogger or conspiracy theorist”, he listed several prominent economists, notably Paul Krugman, Martin Feldstein, Dominick Salvatore, and Rudiger Dornbusch, who had predicted the problems of the Eurozone (Bagnai 2011). Even when speaking at political rallies alongside Salvini, he frequently referred to such well-known scholars, suggesting that the leader of the Lega had just listened to and read a good popularization of the truths discovered by these eminent social scientists. In Florence, on 7 February 2018, Bagnai stated, speaking of Salvini, that “this ‘dangerous extremist’ is a person who is saying a thing that every economist knows and that was said in 1971 by an important left-wing economist, [Nicholas] Kaldor, who was even a Lord, namely that if we do a monetary union before the political one, there will be such economic tensions that we will compromise forever the political union. This is what is happening”. This stance was blended with a periodic mockery of “Sunday epistemologists”, i.e. people without any professional legitimacy casting doubt upon Bagnai’s convictions on the ground that economics’ weak scientific status does not allow him to make predictions about what would happen were Italy to leave or not the eurozone. He thus stated that “whoever says that ‘economics is not a science’ is doing propaganda for the neoliberal project” (Bagnai 2017b).

But Bagnai’s emphasis on the value of first-rate economic knowledge is dwarfed by his growing concern that science can be and in fact is regularly *perverted* in various ways: it can become an unquestioned ideology whose authority is exploited by powerful interest groups to advance their goals against the common good. Bagnai often refers to the case of Mark Hegsted, a nutrition scientist who is notorious for having been accused of minimizing the link between sugar consumption and heart disease due to his funding connections with the sugar industry. The fact that his incriminated paper had been published in one of the most prestigious medical journals, the *New England Journal of Medicine*, was taken by Bagnai as proof that “one can simply doll up a pre-packaged thesis and pass the mythical peer-review”, adding that “a good 90% of the research on the euro is visibly of this type” (Bagnai 2017e). He later added that “in 2017, the literature on the pros [of the euro] is puny and mostly in conflict of interest, being almost entirely funded directly or indirectly by institutions whose survival is linked to the euro” (Bagnai 2017f). The implication is that people should reassert democratic control of certain domains of life that have been de-politicised and handled by experts. This idea emerges clearly in a 2016 exchange that Bagnai had with several other economists on Twitter. These were advocating the creation of “independent agencies for rating public policy proposals”, to which Bagnai retorted that “unless you are a fascist, you should acknowledge that such agencies exist. We use to call them Parliaments, in our old-fashioned democracies” (Bagnai 2016). He ridiculed the whole post-truth discourse and the measures taken in 2017 by the Gentiloni government to fight fake news as being reminiscent of an Orwellian “Ministry of Truth” (Bagnai 2017a). These claims were often combined with harsh denunciations of high-profile economists such as Giavazzi and Alesina acting as “organic intellectuals of the capitalist class”, and of economics correspondents working for mainstream media acting as “hired guns”, “regime’s misinformators”, and “regime’s clowns”. Morally connoted terms such as “treason” and “deception” are common in Bagnai’s characterisation of intellectuals (and elites in general) and channel a sense of antagonism between the people and the elite.

Other perversions of expert knowledge Bagnai chastises include the supposed independence of bureaucracies in general and of central banks in particular, an idea he designates as a “fetish”, “an antidemocratic and ruinous dogma”, whose consequence is keeping governments under the duress of markets whenever they try to redress the hardships of the country through fiscal policy. He often uses the now popular acronym TINA to ridicule the ideology according to which there is no alternative other than staying in the Eurozone and adopt austerity policies (Bagnai 2014e). Journalist and medias cannot be trusted, since they are complicit in the EU technocratic project: in one occasion, he defined journalists as “lackeys of the capital, henchmen of the fascism of public opinion, the cancer of democracy” (Bagnai 2017d). Instead, trust should be accorded to courageous politicians and critical intellectuals, who establish a charismatic connection with their followers using various channels (including blogs and associations) and energise social movements to enhance public participation for more democratic decisions. Accordingly, his discourse often turns prophetic, hinting that he is the custodian of a secret body of knowledge. This exalted attitude is particularly visible in his blog and in social networks, where Bagnai frequently deploys a blunt and outspoken rhetoric, banning and blocking readers who criticise him, and using colourful epithets to insult abstract entities (such as the “*piddino*”, the typical PD voter) as well as concrete individuals.

The tension between Bagnai’s emphasis on the value of scientific consensus in economics and his distrust for many sources of economic knowledge is settled through the concept of “Thescience” [*Lascienza*]. Thescience is for Bagnai and his followers a mockery of true science: non-democratic (as Burioni would say), dogmatic, siding with the powerful. By contrast, true science is democratic and constantly open to doubt. Thus, Bagnai can argue that his blog originated “in the desire to redeem science from her slutty cousin, Thescience” (Bagnai 2017g).

It is hard to tell whether Bagnai’s arguments actually were instrumental to the flourishing of anti-euro positions within the Lega, or whether they should be explained by reference to more profound symbolic associations (such as connecting the euro with globalisation, foreigners, and big banks) and strategic calculations by Salvini and other leaders. A draft of the 2018 government deal between the Lega and the M5S contained a motion to “introduce specific technical procedures of a juridical and economic nature that will allow member states to withdraw from the monetary union, and thus to regain their monetary sovereignty” (Huffington Post 2018).^8^ However, in the final document, signed in May 2018, this paragraph was replaced with a general indication advising to “reconsider, along with the European partners, the structure of European economic governance (monetary policy, Stability and Growth Pact, Fiscal compact, ESM, Macroeconomic Balance [*sic*] Procedure, etc.) which today is asymmetrical and based on the dominance of the market instead of a wider economic and social dimension” (il Post 2018a). Since its appointment, the government has been more interested in confronting the EU over its budget deficit than in setting up procedures to leave the Eurozone. In September 2019, a new government was formed with the support of the M5S and other centrist and left-wing parties, with Salvini’s Lega back in the opposition.

## A Political Sociology of Public Epistemologies

Burioni and Bagnai have become representative figures of the two main epistemological discourses present in contemporary Italian political debates: the science aversion narrative and the science perversion narrative (Table 1 resumes the main features of the two public epistemologies). Although Burioni and Bagnai exploited and reinterpreted ideas already present in the public sphere before their rise to prominence, they also contributed to shaping these narratives by creating associations, disseminating ideas, and coining catchphrases that have become rallying cries for groups struggling for power. Despite the fact that their work focuses, respectively, on vaccines and monetary systems, their discursive products now circulate and are used by their followers and imitators across many different areas of debate. As a matter of fact, Bagnai has made several incursions into the debate on vaccines and has expressed scepticism toward the extension of mandatory vaccination, while Burioni has made various allusions indicating his disapproval of anti-euro economic theories.^9^

**Table 1:** Two public epistemologies in Contemporary Italy

	Science aversion	Science perversion
The purpose of science is	Solving problems	Challenging dogmas
The opposite of science is	Idiocy, arrogance	Ideology, conformity
Falsehood persists because	New technologies are making us dumber	Powerful groups prevent truth from being told
Falsehood is spread by	Arrogant and uneducated people	Mainstream experts and journalists
Truth is revealed by	Accredited scientists	Informed activists
Trust requires	The right credentials	Charisma
Politics is	The struggle between reason and emotion	The struggle between the people and the elites

That Burioni and Bagnai enjoy similar levels of popularity and political power also suggests that public attitudes toward science in Italy cannot be reduced to either irrational hostility and distrust of expertise or uncritical reverence of scientific authority. Instead, these two antithetical attitudes should be conceived as part of two public epistemologies that are used by competing factions in the political struggle. Eyal is right to argue that “what needs to be explained it not a one-sided ‘death of expertise,’ ‘mistrust of experts,’ or ‘assault of science,’ but the two-headed *pushmi-pullyu* of unprecedented reliance on science and expertise coupled with increased suspicion, scepticism, and dismissal of scientific findings, expert opinion, or even of whole branches of investigation” (2019, p. 4). However, it is important to recognise that these two objectively occurring processes are also complemented by a discursive and ideological level, i.e. the level of public epistemologies, that is shaped by these objective processes and shapes them back. Technocratic encroachment and populist anti-intellectualism are first and foremost imagined threats that are discursively constructed by different factions involved in political battles, with the help of intellectuals such as Burioni and Bagnai.

But what exactly are these factions? One possibility is that the two Italian public epistemologies are connected to transformations occurred in recent years in the political landscape of Italy and other Western countries. They could be described, to simplify to the extreme, as being part of the ideology of populist and liberal (or, to be more faithful to Italian lexicon, “reformist”) political forces. Many observers believe that a new opposition, orthogonal to the classic left and right dimension, increasingly structures contemporary political debates across the world. Although the particulars vary sensibly, the diagnosis is similar: we are witnessing a rise of populist movements and parties, which appeal to a homogenous, authentic people rooted in a specific national culture in opposition to unelected administrative bodies, condescending intellectuals, powerful institutions, foreign powers, and elites of various sorts.^10^ The electoral achievements of parties such as the M5S and the Lega in recent years testify to the importance of this trend in Italy. Opponents of populism are a heterogeneous bunch, including social-democrats, “third way” liberals, Christian-democrats, centrists of many sorts, but they tend to be united by a common attraction toward traditional and mainstream ways of doing politics. They value pluralism, the rule of law, civil rights, free markets, international integration, and they tend to view favourably the development of unelected expert bodies, regulatory committees, and independent agencies that have gained a prominent role in shaping public policies in the last decades (Jasanoff 1990, Radaelli 1999).

We could then consider the science perversion narrative as the epistemological facet of Italian populism, and the science aversion narrative as the epistemological facet of Italian liberalism. Burioni nicely illustrates the anti-populist inclination of the science aversion narrative when he offers the analogy between talking about mandatory vaccination and commentating a football match: everyone knows that there are rules in football and that these must be known to be able to talk about it; whereas, when talking about compulsory vaccination, everyone thinks they have a say in the discussion even without knowing the “rules”, i.e. immunological scientific evidence. Thus Burioni, in classic anti-populist fashion, suggests that the gap between the mass of laypeople and highly specialised experts should be widened rather than narrowed: the popular will cannot translate directly into policy but must be mediated by independent scientific institutions. Conversely, Bagnai epitomizes the populist bent of the science perversion narrative when he claims that “a good 90%” of the research on the euro cannot be trusted because it is funded by pro-EU organisations, or when he chastises the “fetish” of central bank independence. This is what many scholars have identified as a key piece of the populist repertoire, and what Brubaker designates as “antagonistic repoliticization”, i.e. “the claim to reassert democratic political control over domains of life that are seen, plausibly enough, as having been depoliticized and de-democratized, that is, removed from the realm of democratic decision-making” (2017, p. 364). Further research could investigate whether the two narratives are generalisable to other cultural contexts, and whether the link between public epistemologies and political commitments stands closer scrutiny.

Speaking of the science perversion narrative as one of the possible epistemologies of populism might sound strange to many. There is a long-lived and heterogeneous body of literature arguing that populism puts experience, what people see with their own eyes, over knowledge, i.e. facts and theories about the world (Hofstadter 1963, Saurette & Gunster 2011; Speed & Mannion 2017; Waisbord 2018). In this perspective, a populist public epistemology is a contradiction in terms, as populism operates according to an “anti-epistemology” denying any value to abstract reasoning. The Italian case of Bagnai suggests instead that populist movements include intellectuals who develop sophisticated theories about the nature of truth, the role of scientific institutions in society, and the methods that should be followed to acquire knowledge of the natural and social worlds. Rather than denying any value to the concept of truth, as suggested by authors claiming that we have entered an age of post-truth politics, populist movements can advocate and produce what Ylä-Anttila calls “counterknowledge”, hence professing a “belief in truth achievable by inquiry, not by mainstream experts but alternative ones” (2018, p. 356). Furthermore, populist movements are in all likelihood internally differentiated in various groups with distinctive “truth orientations”, i.e. attitudes (rather than the coherent narratives analysed here) toward scientific knowledge and expertise (Ylä-Anttila 2018, p. 379). Applying this insight to the Italian case, it might be argued that Bagnai mainly represents and addresses the most highly educated sections of the people identifying with and voting for the Lega. Overall, my analysis of public epistemologies in Italy joins other recent works suggesting that scholars should pay more attention to the complex and multifaceted cognitive and intellectual dimensions of populist, nationalist, and right-wing movements (Merriman 2019, Panofsky and Donovan 2019).^11^

A similar critique could be levelled against my account by those who believe that perceptions of and approaches to science cannot be reduced to either quasi-technocratic or quasi-populist ones, on the grounds that this is a false dilemma. Indeed, the two Italian public epistemologies are reminiscent of two of the three idealised conceptions (or “waves”) of the relations between science and society identified by Collins and Evans (2017). According to the first conception, which runs from the early twentieth century up until the 1960s, “science was unquestionably the pre-eminent form of knowledge-making” and “its knowledge was absolute and universalistic,” while the second conception, which emerged in the 1960s, “provided a powerful argument against technocracy by showing how expert advice rested on a sea of social assumptions” leading to “arguments in favour of the democratization of science, and of expertise more generally.” Collins and Evans refer to the academic discipline of science studies, but write that at least the first conception “informs many popular representations of science” (2017, p. 17–18). Applying this scheme to the Italian case, it is easy to see how Burioni’s science aversion narrative implicitly draws from Wave One theories, while Bagnai’s science perversion narrative draws from Wave Two challenges to the objectivity of expert advice. In a typical synthetic move, Collins and Evans then suggest that a new conceptualisation of science-society relations is needed, and theorise a Wave Three that combines expert authority and democratic accountability, while rejecting both “technological populism” and technocracy. Here, too, there are no compelling signs that such higher synthesis obtains in contemporary Italian politics. While it is possible that a third public epistemology, alternative to the science aversion and perversion narratives, will be developed by prominent intellectuals, perhaps closely associated with new radical left-wing movements, nothing quite like that exists at the moment. One might argue that some intellectuals have criticised technocratic depoliticisation while also resisting populist capture, especially in connection with protests against major infrastructure projects such as the Turin–Lyon high-speed railway (Pellizzoni 2011, Chiaramonte 2019), but none has acquired the influence and power that Burioni and Bagnai enjoy.

Furthermore, Burioni and Bagnai have tried in their own way and to different degrees to synthetize different perspectives, integrate critiques and respond to counterarguments. Burioni admitted that individual scientists can be wrong, while Bagnai argued that a Nobel prize signals that an economist deserves to be heard. Each of the two might claim that his views are complex and well-rounded, while his opponent’s views are just the expression of one-sided fanaticism. In reality, what can be observed is just a conflict between two opposite positions, without anything really suggesting that one side is overcoming the tension between expert authority and democratic participation. As much as this can be considered a false dilemma, it is what recent Italian politics has produced in terms of public epistemologies.

## Footnotes

1 My approach is symmetrical and inclusive regarding what should count as epistemology. This is far from being an obvious choice. Reading my descriptions of public epistemologies below, some readers might think not only that they are fundamentally flawed, but that they should not be considered as epistemology at all. Similarly, in debates about secularism and the existence of God, critics of religion often insist that biblical faith “is *not* an epistemology”, since it is not “a way of knowing”, but “a way of trusting” (Barnett 2018). What counts as an epistemology is a contested convention, and often this is because attributing the status of epistemology to someone’s ideas would be seen by their opponents as conferring them an undeserved privilege. The approach I advocate is instead inclusive: if something possesses some family resemblances with the things that are generally associated with the concept of episteme, then we should include rather than exclude it from our studies.

2 Public epistemologies should thus be distinguished from “epistemology” as a specific philosophical branch, and this is one reason why the plural form (“epistemologies”) should be favoured.

3 See Camera dei Deputati 2018a: 59, 109, 119, 130, 132, Camera dei Deputati 2018b: 53, 83, 238, 246, 251, 282, Senato della Repubblica 2018: 39, 45.

4 Although not exactly mapping onto my categories, Bucchi’s (2010) analysis of the antagonism between a pro-science faction and anti-science faction in contemporary Italy already pointed to the ubiquity of public debates about science and underlined the tendency of both sides to reify both science and society as discrete and autonomous entities.

5 Many scholars have recently emphasised the importance of “social” or “Web 2.0” websites for understanding how intellectuals operate and ideas spread in contemporary societies (e.g. Dahlgren 2012; Drezner 2017; Bacevic 2018, Carrigan 2019). My study bears witness to this: one of the two intellectuals I will consider has tweeted 183,000 times from October 2012 to February 2019, averaging a staggering 80 tweets a day. Intellectuals do spend *a lot* of their time online today. Accordingly, I do not see any major objections against analysing debates on Twitter, describing the content of Facebook pages, or analysing the comment section of a weblog. However, studying these things poses specific challenges: online users can very easily edit and delete the content of their publications (whether it is a blog post, a comment, or a whole webpage), without any notice to the external observer, online content can be counterfeited easily, and users and whole websites can be created to imitate one’s intellectual opponents in a distorted way, to bring them into disrepute. Extreme caution should therefore be exercised when studying online debates.

6 This stance resonates with a general trend that has made the educated public and scientists themselves speak of science in a cool and down to earth fashion: think of Neil deGrasse Tyson saying that “Einstein was a badass,” Matt Damon’s astronaut in “The Martian” vowing to “science the shit” out of problems, or the popular website tellingly called “I fucking love science”. See Semley (2017) for more examples and a brief critique of this trend.

7 The most noteworthy publication was Bruno Amoroso’s *Euro in bilico* (2011)*.*

8 Apart from Bagnai, this formulation was probably demanded by Paolo Savona, an anti-euro economist and high civil servant whose nomination as minister of the economy was successfully opposed in May 2018 by president Sergio Mattarella.

9 The parliamentary speech quoted above about science being the “continuation of politics by other means” is Bagnai’s. By and large, Bagnai has expressed his vicinity toward intellectuals and researchers who have been critical of vaccination laws, claimed that the PD addressed the issue of vaccination in a “hysterical way” (FirenzeToday 2018), and hinted that the whole debate on vaccines is a “giant weapon of mass distraction” (Bagnai 2017c). Burioni’s regularly intervenes in heated online exchanges siding with pro-euro economists and politicians, adding remarks such as “I am perfidiously enjoying it: for two years I have thought that idiots would show interest only in immunology, instead I am pleased to see that even economists have their fair share” (Burioni 2018).

10 This vast literature includes such differentiations as high versus low (Ostiguy 2017), national populism versus neoliberalism (Eatwell & Goodwin 2018), illiberal democracy versus undemocratic liberalism (Mudde 2015), somewheres versus anywheres (Goodhart 2017), globalists versus nationalists (Haidt 2016), and rationalists versus intuitionists (Oliver & Wood 2018).

11 The claim that Burioni and Bagnai represent the epistemological version of the political opposition between liberalism and populism might raise some eyebrows on the grounds that it neglects radical left-wing movements and obfuscates the right-left distinction under the vague notion of populism. However, and as unfortunate as it might be, radical left parties have enjoyed very little support in Italy during the decade between 2010 and 2020, a fact all the more striking for a country that has been home of the strongest communist party of the West in the post-war period (Broder 2020). Besides Bagnai, many media intellectuals, such as Marxist-Hegelian philosophers Paolo Becchi and Diego Fusaro, progressively rejected the left-right opposition altogether during this period, often aligning with the Lega and the M5S and having contacts with explicitly neo-fascist groups (Bianchi 2017b). Other intellectuals traditionally close to the left have similarly dismissed old divides by joining the reformist camp, such as former communist philosopher Massimo Cacciari commending Monti’s efforts to liberalise the market and imploring him to run for Prime Minister (Cacciari 2012). Of course, many Italian intellectuals still identify with the radical left and regularly denounce both the crass bigotry of the populists and the elitism of the reformists. However, they often advance epistemological arguments against science perversion that are similar to those that Bagnai has been marshalling since 2011. For interesting perspectives from the left see Tomatis (2015), Dalla Riva (2019), and Sandal (2019).
